# The Effect of Gambling on Saving Behaviour in Australia

**DOI:** 10.1007/s10899-025-10409-8

**Published:** 2025-06-17

**Authors:** Opoku Adabor

**Affiliations:** School of Economics, Finance, and Marketing (EFM), RMIT University, Melbourne, Australia

**Keywords:** Savings behaviour, Gambling, Locus of control, Social capital and Australia

## Abstract

Although gambling can be a form of entertainment for some, it is commonly associated with social, psychological, financial, and physical harm. To reduce the adverse effects of gambling, it is important to examine the implications of gambling for policy formulation. In this study, we examine the causal impact of gambling on savings behaviour using a sample of gamblers from Australian household panel data for the period of 2015 and 2018. Our results show that gambling is associated with poor savings behaviour. This result is robust to different measures of gambling, eight different sub-groups and four gambling risk statuses. Our mediation results show that social capital and locus of control are mechanisms through which gambling influences saving behaviour.

## Introduction

Saving behaviour refers to how households or individuals manage their income by setting aside a portion for future use rather than spending it immediately (Furnham, 1999; Carroll et al., 2000). This behaviour can be consistent, irregular, passive or active (Cronqvist & Siegel, 2015). People often save to create a financial buffer for unexpected events like medical emergencies, and job loss and to be financially stable in the long run (Horioka & Watanabe, 1997; Stix, 2013). While psychological, cultural and social norms factors such as self-control, spending habits, future orientation, and risk tolerance have been cited as the leading causes of saving behaviour (Tanaka & Murooka, 2012; Fuchs-Schündeln et al., 2020; Srivisal et al., 2021), other studies have identified economic, environmental, and financial factors such as income, interest rates, and inflation as important contributors to savings behaviour (Koskela & Virén, 1982; Ozcan et al., 2003; Jumena, 2022; Mohanta & Dash, 2022). For instance, risk-averse people tend to save more, while people with impulsive spending tendencies save less (Klonner, 2003; Bommier et al., 2012; Levenko, 2020). Although the extant literature has identified numerous factors that influence gambling, surprisingly, little or no evidence exists on how gambling impacts saving behaviour.

Gambling defined as the act of wagering money or something of value on an event with an uncertain outcome can impact savings behaviour in several ways. First, excessive gambling often leads to financial losses (Mathews & Volberg, 2013; Koomson et al., 2022; O’Sullivan, 2023). Individuals who regularly lose money to gambling may find themselves with less disposable income, which makes it difficult to save periodically. Second, gambling can disrupt plans for saving by misallocating financial resources that might otherwise go toward savings to gambling. This misallocation can also disrupt financial stability, negatively impacting saving behaviour (Graydon et al., 2019; O’Sullivan, 2023). Third, gambling addiction can overshadow the desire or ability to save. Individuals addicted to gambling may prioritize gambling over other financial responsibilities including savings, leaving little room for long-term wealth accumulation. Additionally, financial loss associated with gambling causes stress and anxiety (Buchanan et al., 2020; Koomson et al., 2022), making it difficult to maintain a healthy saving behaviour. Thus, the emotional toll linked with gambling can reduce motivation to save or engage in sound financial planning.

As gambling becomes more accessible through online platforms and physical gambling establishments (Sirola et al., 2018; Chóliz, 2016), it raises concerns about how individuals’ spending patterns may change, particularly regarding their ability to save money. Individuals who engage in gambling activities are less likely to prioritize savings, if they experience impulsive spending, or if they are more prone to financial distress due to gambling losses (O’Sullivan, 2023; Koomson et al., 2022). Excessive gambling may lead to financial instability, decreased savings, and an overall decline in economic well-being (Mathews & Volberg, 2013). This study aims to explore how gambling behaviours influence individuals’ savings decisions and whether gambling habits contribute to reduced or delayed savings, ultimately affecting long-term financial security. The study also explores social capital and locus of control as mechanisms through which gambling behaviour impacts saving behaviour.

Indirectly, gambling can impact saving behaviour via social capital. Empirical studies have shown that gambling, especially problem gambling can strain relationships and lead to conflict, especially if the gambler’s behaviour leads to financial instability (Holdsworth et al., 2013; Dowling et al., 2016; Carr et al., 2018). The breakdown of relationships weakens social cohesion, which is known to improve saving behaviour. Thus, gamblers may be unable to maximize social cohesion to better their saving behaviour (Griswold & Nichols, 2006). In some extreme cases, problem gambling can lead individuals to rely on others to help cover living expenses or pay debts (Latvala et al., 2021), further reducing their capacity to save.

Locus of control is also an important pathway through which gambling impacts savings behaviour. Individuals can be either external or internal on locus of control. Individuals with internal locus of control believe they have control over their lives and thus their own decisions, efforts, and abilities determine the results in their lives (Cobb-Clark, 2015). Individuals with an external locus of control believe that their life events are influenced by external forces, such as luck, fate, or other people, and that they have little control over the outcomes (Furnham, 1986). Individuals with internal locus of control are more likely to participate in gambling frequently because they believe that their strategies, decisions, or abilities can influence the outcome. Increased gambling participation leads to financial stress (Koomson et al., 2022), impacting saving behaviour. Individuals with an external locus of control are more vulnerable to developing gambling problems because they may not take responsibility for their behaviour. Once they are caught up with gambling, they may feel powerless to stop gambling, believing that their fate is out of their hands. This can lead to continued gambling despite negative consequences such as financial loss and poor saving behaviour (Mathews & Volberg, 2013).

 Our focus on Australia is important for at least two reasons. First, Australia is one of the developed countries with a high incidence of gambling. Every year, the country spends approximately 226 billion dollars on gambling activities and has the highest gambling loss per capita in the world, standing at approximately AU$1300 per adult as of 2016/2017(Letts, 2018; Baako et al., 2024 ). Further, the country lost nearly AUD 25 billion on legal forms of gambling in 2018/2019 (Letts, 2018; Baako et al., 2024 ). Each month, nearly 35% of adult Australians spend money on gambling, with betting being the highest. Second, there has been a persistent fluctuation in personal savings over the years in Australia. For instance, between 1959 and 2024, personal savings in Australia averaged at 9.26%, which is relatively lower compared to other developed countries like Switzerland and Canada (Australian Bureau of Statistics [ABS], 2024 ). The country recorded its lowest personal savings rate in 2006, at -2.40% (ABS, 2024). Additionally, the country’s household saving ratio decreased to 2.5% from 3.8% between 2022 and 2023 (ABS,2024).

This study makes several contributions to the literature. First, it is the first study to examine both direct and indirect relationships between gambling and savings behaviour. Previous studies have examined other aspects of savings behaviour (Lunt & Livingstone, 1991; Niculescu-Aron & Mihăescu, 2012; Jumena, 2022), without considering the impact of gambling. For instance, Jumena, (2022) found that savings decisions are influenced by factors such as financial service, job profile, self-preference, financial goals and others. Murendo and Mutsonziwa (2017) found that financial literacy positively influences savings behaviour of individuals in both rural and urban areas. Browning (2000) found that the level and composition (portfolio) of savings depend on the household’s income distribution. We differ from this strand of the literature in that we focus on how gambling influences savings behaviour using a large, nationally representative sample from Australia and adopt several measures of gambling. Given the adverse outcomes associated with gambling, our study not only contributes to a growing body of literature on the implications of gambling (Tan et al., 2010; Koomson et al., 2022, Fiedor, 2024; Griffiths, 2024), but add to implementing strategies to reduce gambling participation in Australia.

Our study is closely related to those that have examined the relationship between gambling and saving. For instance, MacDonald et al. (2004) found that the amount spent on gambling reduces savings for retirement. Holdsworth et al. (2013) found that individuals whose partners are problem gamblers experience a loss of their savings. In Singapore, Mathews and Volberg (2013) find that problem gambling reduces the savings of family members. Contrary, Herskowitz (2021) found that randomized savings treatment decreases demand for betting via unmet liquidity needs and saving ability. Unlike these studies, which focus on saving, we focus on saving behaviour, which differs from savings. Savings behaviour refers to the patterns, decisions, and habits that influence how and why individuals save money, while Savings refers to the actual amount of money that an individual, household, or organization sets aside for future use (Carroll et al., 2000; Furnham, 1999). Additionally, we go beyond these studies to examine locus of control and social capital channels for the link between gambling and savings behaviour, contributing to studies that have examined the direct impact of social capital and locus of control on saving behaviour (Lunt & Livingstone, 1991; Di Falco & Bulte, 2011; Bucciol & Trucchi, 2021).

## Theoretical and Empirical Literature Survey

Economic theories that explain savings behaviour and gambling behaviour delve into the ways individuals make decisions about allocating resources, particularly how they manage risks and time preferences. For instance, the Life-Cycle Hypothesis (LCH) proposed by Ando and Modigliani (1963) suggests that individuals plan their savings and consumption based on their expected lifetime income. This theory explains that people smooth their consumption over time to maintain a stable standard of living throughout their lives. Contrary, the Permanent Income Hypothesis (PIH) introduced by Friedman (1957) suggests that people save and consume based on their expected long-term (permanent) income rather than their temporary or current income. According to the PIH, consumption is driven by permanent income, not transitory income changes, which leads individuals to smooth their consumption and save more during periods of higher income or economic stability.

The prospect theory, developed by Kahneman and Tversky (2013), explains why people engage in gambling behaviour, especially under conditions of risk and uncertainty. According to the theory, individuals value gains and losses differently: they are more sensitive to potential losses than equivalent gains (loss aversion). This can lead people to take greater risks to avoid or recover from perceived losses (e.g., chasing losses in gambling).

The theory of mental accounting, developed by Thaler (1985), explains the relationship between saving behaviour and gambling. This theory refers to the cognitive biases individuals have when they treat money differently based on subjective criteria (e.g., the source of the money). For example, someone might be more willing to spend or gamble “windfall” money (e.g., lottery winnings, tax refunds) than to dip into savings or earned income. This is relevant for both gambling and savings decisions because it shows how people separate money into mental “buckets,” which can lead to irrational financial behaviours.

Empirically, many studies have been conducted to unearth the factors that influence savings behaviour. Such factors include job security, self-efficacy, access to financial services, personal wealth, individuals’ needs, cultural and social norms, age, financial goals and others (Jumena, 2022; Murendo & Mutsonziwa, 2017; Browning, 2000). For instance, Cronqvist and Siegel (2015) found that savings behaviour is genetically correlated with income growth, smoking, and obesity. Cruz et al. (2025) found that personal values towards goals and survival needs are positively correlated with savings behaviour. One of the factors that has received much attention is financial literacy. Existing work on financial literacy as a determinant of savings behaviour cuts across sociology, economics, and psychology. Within this strand of the literature, most studies found that enhanced financial literacy is one of the important factors explaining savings and financial behavioural changes (Murendo & Mutsonziwa, 2017; Alshebami & Aldhyani, 2022; Sayinzoga et al., 2016).

Besides these factors identified to influence savings behaviour, A growing body of literature has identified social capital and locus of control as essential factors that shape people’s savings behaviour (Cole et al., 1992; Yayeh & Demissie, 2025; Bucciol & Trucchi, 2021). On one hand, social capital was found to positively influence savings behaviour by shaping trust, norms, and social networks that affect how people make financial decisions (Yayeh & Demissie, 2025). On the other hand, individuals who are internal on locus save more and more often, while those who are external on locus of control save less and less often (Bucciol & Trucchi, 2021).

The gambling literature has identified the impact of individuals’ gambling participation on numerous outcomes. Although this literature has identified that gambling impacts wealth, household and disposable income, emphasis is increasingly placed on the effect of individuals’ gambling behaviour on health, social, sociocultural, socioeconomic and environmental factors (Fiedor et al., 2024; Baako et al., 2024; Koomson et al., 2022; Griffiths, 2024). For instance, Baako et al. (2024) found that gambling reduces the likelihood of owning a home. Churchill and Farrell (2018) found that gambling behaviour increases the risk of depression, with online gambling posing a significant mental health risk compared to gambling at venues or outlets. Gambling has also been shown to impact locus of control and social capital. Over time, frequent gambling can condition people to think that luck, not effort, determines success (von der Heiden & Egloff, 2021; Clarke, 2004). This can shift their locus of control externally, especially if they experience random wins. On social capital, gambling has been found to negatively impact social capital by eroding trust, weakening social networks, and disrupting the norms and values that hold communities together (Griswold & Nichols, 2006; Reith & Dobbie, 2011). However, in some specific contexts, it can also temporarily build certain types of social bonds (Bond et al., 2024).

**Fig. 1:**
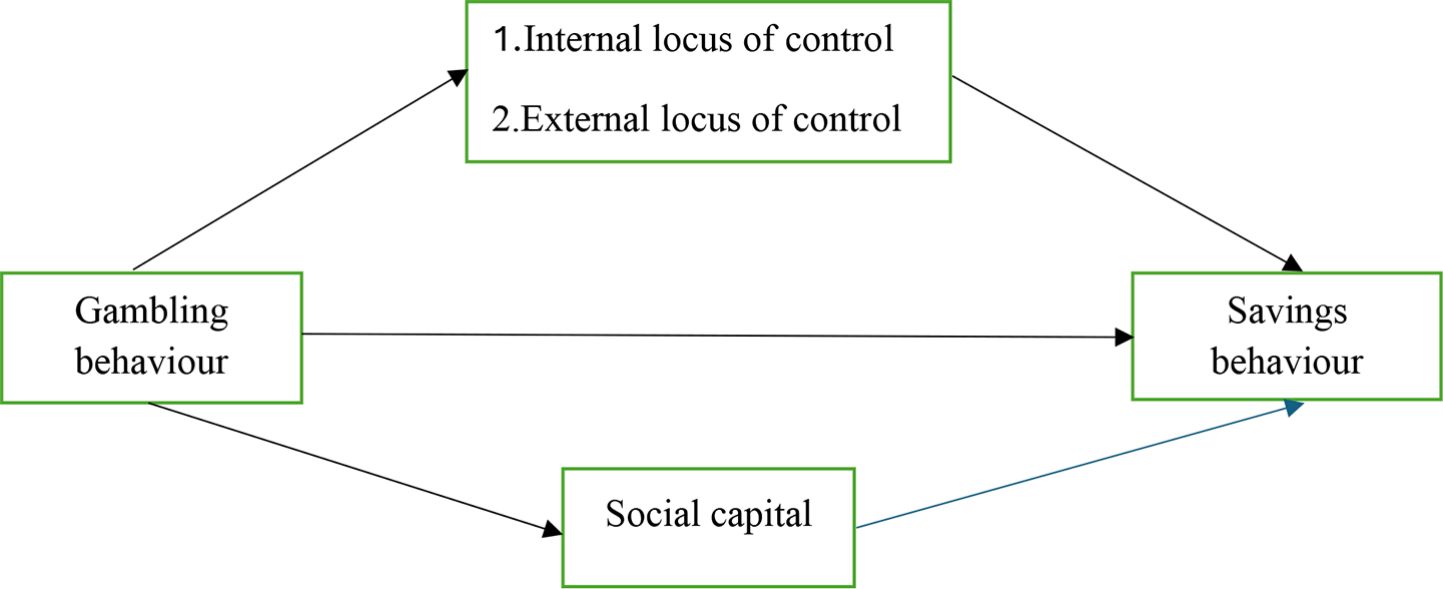
Conceptual relations between gambling and savings behaviour

Overall, the Mental accounting theory of behavioural economics explains the direct relationship between gambling and saving behaviour. From an empirical perspective, gambling has an indirect impact on saving behaviour via its effect on social capital and locus of control. Therefore, we built a simple conceptual model in Fig. 1, portraying three main objectives based on theory and literature: (1) Individuals engaged in gambling are less likely to engage in traditional saving behaviours, (2) Gambling nudges people to be either inter or external on locus of control, which influences shapes people’s savings behaviour overtime and (3) Gambling weaken social capital to impact saving behaviour negatively.

## Data and Variables

The study uses the Household, Income and Labour Dynamics Survey in Australia (HILDA). The HILDA survey is a nationally representative household data conducted in Australia. This survey collects information on several outcomes, including education, energy, gambling, health, and job market dynamics. This information is gathered from an average of 17,000 across selected households. The HILDA survey began in 2001 and has produced 23 waves (Watson & Wooden, 2012). This study is based on two waves conducted in 2015 and 2018 with a response rate of about 67.1% and 63.6%, respectively. We use these two waves because information on gambling-related activities is available in only these two waves. Although the information on saving behaviour is available in these waves and other waves, we cannot use more than two waves, as questions relating to households’ participation in gambling activities are not available in the other waves. After merging the two waves and at the same time adjusting for missing data in both outcome and control variables, the maximum number of observations for our regression model is 16,353.

### Saving Behaviour

There are various forms of savings behaviour, which can differ based on the individual’s goals, financial discipline, and approach to money (Browning, 2000; Nyhus, 2017). The first one is consistent saving behaviour which involves regularly setting aside a certain portion of income over time, such as through automated deductions to savings accounts or retirement funds (Cronqvist & Siegel, 2015). The second form of saving is irregular or opportunistic saving which generally involves saving extra income or after an unexpected financial windfall, like a tax refund or a bonus at work (Brown & Taylor, 2016). The third form of saving is passive savings which involves saving unintentionally or automatically, such as when employers deduct retirement savings from paychecks before the money reaches an individual’s hands (Van Veldhoven & Groenland, 1993). Active saving is the fourth form of saving behaviour which involves actively monitoring your spending and saving habits, often tracking your expenses and making intentional decisions to increase savings (Nyhus, 2017).

In this study, we employ two main measures of saving behaviour based on information from the HILDA Survey. Precisely, we measure saving behaviour using a questionnaire in the HILDA survey that asked respondents about their savings habits. The HILDA survey asked respondents how often they save and provided the following responses: [1] Don’t save: usually spend more than, [2] Don’t save: usually spend about all [3] Save whatever is left over -no regular savings [4] Spend regular income, save other income [5] Save regularly by putting money aside. In this case, our first measure of savings behaviour is as a binary variable set equal to one if respondents selected “[5] Save regularly by putting money aside” to respond to this question. This shows that respondents save regularly, reflecting consistent savings. Our second measure of savings behaviour is a binary variable set equal to one if respondents selected “Save whatever is left over -no regular savings” to respond to this question. This shows that respondents portray irregular or opportunistic saving behaviours.

### Gambling

Consistent with previous studies (Koomson et al., 2022; Adabor, 2023; Ackermann et al., 2024), we use the problem gambling severity index (PGSI) as a measure of gambling. To compute the PGSI, we use information from the HILDA survey that asked respondents about their gambling behaviour, participation, and the adverse impact that they experienced from gambling in the past 12 months. Specifically, the HILDA survey poses nine different gambling questions, and a four-point scale is used to rate responses to the nine questions (Table 1A in the appendix lists all these gambling questions). The responses to these questions are on a scale of 0 to 3, where 0 means never, 1 means sometimes, 2 means sometimes and 3 means almost always. The gambling severity index is generated by summing these responses to produce a PGSI score ranging from 0 to 27. A high PGSI score indicates an increasing severity of problem gambling. Thus, increasing the PGSI score suggests a higher risk of gambling.

As a robustness check, we use gambling risk as an alternative measure of gambling. Gambling risk is a 1–4 gambling risk scale computed based on the PGSI. Four different risk statuses are identified: (1) non-problem gamblers (these individuals were assigned a PGSI score of ‘0’, suggesting that they have never experienced the detrimental effect of gambling over the past years or did not engage in gambling behaviour); (2) low-risk gambler (these individuals were assigned a PGSI score of ‘1 or 2’, suggesting that they engaged in gambling for fun, and hence, gambling is not likely to have a severe impact on them. They may rather benefit from gambling); (3) moderate-risk gamblers (these individuals were assigned a PGSI score of ‘3 to 7’, suggesting that they engage in gambling moderately and are likely to experience the detrimental effect of gambling moderately); (4) problem gamblers (these individuals were assigned a PGSI score of ‘at least 8’, suggesting that they are gambling adductors and often experience the harm linked with gambling). Based on these four categories of risk gamblers, we developed a gambling risk scale ranging from 1 to 4, where an increasing score suggests a high risk of gambling. This means of measuring gambling risk is commonly used in empirical works (Carneiro et al., 2020; Koomson et al., 2022).

### Mediating Variables

This study considers locus of control and social capital as mediating variables for the relationship between savings behaviour and gambling. In this section, we demonstrate how these mediating variables are measured.

The HILDA survey measures locus of control (LoC) with the Psychological Coping Resources of the Mastery Scale (Pearlin & Schooler, 1978). Pearlin and Schooler (1978) developed the Mastery Scale which is a psychometric validated instrument that is based on the following questions: ‘(1) I have little control over the things that happen to me; (2) There is no way I can solve some of the problems I have; (3) There is little I can do to change many of the important things in my life; (4) I often feel helpless in dealing with the problems of life; (5) Sometimes I feel that I’m being pushed around in life; (6) What happens to me in the future mostly depends on me; and (7) I can do just about anything I set my mind to do’. Based on previous studies we created a composite indicator of LoC that combined all seven questions such that seven depict internal LoC and one indicates external locus of control (Cobb-Clark et al., 2014; Churchill & Smyth, 2021). Studies have demonstrated that LoC is exogenous, and stable for the working class (Buddelmeyer & Powdthavee, 2016). On this empirical basis, we restrict our sample to adults aged between 21 and 59 to capture the working class.

To measure social capital, we use information on the frequency of people at which individuals gather with friends or family socially. Precisely, we use the HILDA survey question: “In general, about how often do you get together socially with friends or relatives not living with you?” The response to this question is on a seven-point scale where one represents ‘every day’ and seven represents ‘less than once every three months. In line with previous studies (Churchill et al., 2023a, b), we construct a binary variable set equal to one to capture respondents who socially gather with friends or family more than once a month and zero if otherwise.

### Control Variables

In line with the literature on the determinants of saving behaviour and gambling (Farrell & Fry, 2021; Koomson et al., 2022; Adabor, 2023), we control for a rich set of variables to minimise omitted variables bias. Including these control variables is important because they help isolate the impact of gambling on saving behaviour, control for confounding factors, and improve the accuracy of our prediction. Here, we include age, employment status, debt servicing, household size, marital status (separated or widowed), household income (in the log), gender (Female), and educational status as control variables in model (1). Table 2A in the appendix provides detailed descriptions and summary statistics of all these control variables.

## Methodology

To obtain a quantitative impact of gambling on savings behaviour, we estimate the following saving behaviour equation:

1 $$\:{SB}_{ijt}={\delta\:}_{0}+{\delta\:}_{1}{GB}_{ijt}+\sum\:_{m}{\beta\:}_{m}{X}_{n,ijt}+{s}_{r}+{w}_{t}+{Y}_{t}+{\epsilon\:}_{i}$$

Where $\:{SB}_{ijt}$ captures the savings behaviour of an individual$\:i$ living in household $\:j$ whereas $\:{GB}_{jt}$ is the gambling behaviour of individual $\:i$ living in household $\:j$. Here, the individual $\:i$ is mainly a gambler from the general population who has a savings account. This individual is diagnosed with or self-reporting gambling addiction or high-risk gambling behaviour but holds one or more savings accounts. $\:{X}_{n}$ is a set of covariates while $\:{\epsilon\:}_{i}$ represents the error term. $\:{s}_{r}$ and $\:{w}_{t}$ indicate state and wave fixed effects.$\:\:t$ represent time period, covering 2015 and 2018. $\:{Y}_{t}$ represent year-fixed effects. Controlling for these fixed effects is relevant because they account for permanent differences across states, waves and time-varying aggregate trends that influence saving behaviour. To produce baseline results, we applied a linear probability model to estimate Eq. (1) given the binary nature of the outcome variables and to ease the interpretations of the results (note that applying a logit model yielded similar results regarding the direction of the effect).

Endogeneity may be a problem in the baseline results due to measurement errors, omitted variables bias and reverse causality. Measurement errors may occur because respondents may fail to provide accurate responses. Omitted variable bias may happen because we cannot control for all variables affecting saving behaviour and gambling. For instance, we are unable to control for gambling addiction which impacts individuals’ gambling behaviour significantly. While gambling affects saving behaviour, individuals who do not save consistently may use gambling as a means to earn income or money, leading to a reverse causality issue.

To address the endogeneity problem, we use an instrumental variable that leverages on an external instrument to address endogeneity. The main identification here is to obtain exogenous variation in the endogenous variable using the external instrument. In line with previous studies (Farrell & Fry, 2021; Koomson et al., 2022; Baako et al., 2024), we use the number of electronic gaming machines as an external instrument. This instrument is appropriate because it satisfies two conditions: (1) it correlates with gambling and (2) it is orthogonal to the error term in Eq. (1). Individuals’ participation in gambling activities in an area depends on the number of gambling machines available in the area. Thus, a typical proxy considered for gambling is the number of gambling machines available in a geographical area. Hence, there is a strong correlation between gambling behaviour and the number of gambling machines. The instrument is orthogonal to Eq. (2) because the number of gambling machines can impact individuals saving behaviour via its impact on gambling. For instance, the increased number of gambling machines in an area gives more opportunities for individuals to engage in gambling because it makes gambling more attractive, covenants and easily accessible. Further, the availability of gambling machines can lead to excessive gambling, especially in an environment where individuals can gamble without significant social stigma. The increase in gambling participation resulting from the availability of gambling machines will impact gamblers saving behaviour over time.

We complement the traditional instrumental variable strategy with the Lewbel (2012) instrumental estimation technique that does not rely on satisfying the exclusion restriction but rather constructs an internal instrument to address the endogeneity problem. The approach constructs an internal instrument leveraging on the presence of heteroskedasticity in the data. The approach combines internal and external instruments in a case where the external instrument is weak to increase the predictive power of the external instrument. In line with previous studies (Koomson et al., 2022; Adabor, 2024a, b), we aim to use this approach for robustness checks to ensure more reliable estimates of the model parameters.

Overall, this study applies various panel data methods to estimate the effect of gambling on savings behaviour. The use of panel data approaches is important because it helps us to track changes in individual behaviour over time, which is crucial when studying how gambling behaviour affects saving patterns. Savings and gambling are not one-off decisions; they evolve. Someone may start gambling moderately, but over time, this could impact how much they save monthly or yearly. Second, the use of panel data methods helps control for time-invariant individual traits that could bias the relationship between gambling and saving behaviour.

## Results

Table 1 provides the baseline results for the relationship between gambling (i.e. PGSI) and saving behaviour. Precisely, columns 1 and 2 present the results of the impact of PGSI on saving regularly and columns 3 and 4 report the results of the impact of PGSI on saving leftover. Columns 1 and 3 have no control variables while columns 2 and 4 have control variables. Across all the columns in Table 1, the results show a negative relationship between the gambling severity index and saving behaviour and this outcome is higher for models without control variables. Specifically, the results in columns 1 and 2 show that a unit increase in PGSI score reduces the likelihood of saving regularly by 12.5% points and 10.6% points, respectively. In columns 3 and 4, a unit increase in PGSI score reduces the likelihood of saving leftovers by 7.2% points and 6.9% points, respectively. Overall, the results show that gamblers may exhibit poor saving behaviour, consistent with existing studies that have examined the relationship between gambling and savings (Herskowitz, 2021; Holdsworth et al., 2013; Mathews & Volberg, 2013). The economic intuition behind our finding is that gamblers often display present-biased preferences and thus they heavily favour immediate rewards over future benefits. Gambling offers instant excitement and the possibility of a quick payoff. Saving, by contrast, requires delayed gratification, sacrificing now to benefit later. This behavioural bias reduces the motivation to save regularly. Additionally, gamblers may exhibit poor saving behaviour because frequent or compulsive gamblers may chase losses, continuing to gamble in hopes of recovering past losses. This creates a financial downward spiral, draining income or savings to cover debt. Any extra income (that could be saved) often gets redirected toward gambling or debt.

The results from the control variables also provide additional insights. For instance, the results show a positive relationship between various educational statutes and saving behaviour, in line with previous studies (Bhat et al., 2024; Zhang et al., 2003). This effect is bigger for higher levels of education such as having a bachelor’s degree and diploma, indicating individuals with higher levels of education exhibit better saving behaviour. Educated people exhibit good savings behaviour because they are more likely to understand why saving is important, how to budget, and where to invest money. They also tend to recognize the long-term value of saving versus short-term spending. Being unemployed correlates negatively with saving behaviour and owning a home is positively correlated with saving behaviour. Various marital statuses included in the model correlate negatively with saving behaviour, except for never married. For example, being a window reduces the likelihood of saving regularly. Also, having a long-term health condition and the total number of children negatively correlate with saving behaviour.

**Table 1:** Saving behaviour and gambling-Baseline results

	(1)	(2)	(3)	(4)
	Save regularly	Save regularly	Save leftover	Save leftover
PGSI	-0.125***	-0.106***	-0.072***	-0.069***
	(0.014)	(0.014)	(0.010)	(0.010)
Observations	16,384	15,295	15,500	16,353
R-square	0.148	0.282	0.302	0.292
Control variables	No	Yes	No	Yes
State Fixed Effects	Yes	Yes	Yes	Yes
Wave Fixed Effects	Yes	Yes	Yes	Yes
Year Fixed Effects	Yes	Yes	Yes	Yes

Note: We include relevant control variables in all regression. Full results can be found in the appendix Table 2 A. Standard errors in parentheses

*** *p* < 0.01, ** *p* < 0.05, * *p* < 0.1

Table 2 reports the results for both two-stage least square(2SLS) and Lewbel (2012) 2SLS approaches. Panel A results are from the traditional 2SLS while results in panel B are from Lewbel (2012) 2SLS with both internal and external instruments. In both panels A and B, the first stage results show a positive relationship between the number of gambling machines and PGSI, indicating that gambling participation rate goes higher when gambling machines in an area increase. Also, the F-statistics for all the estimates are above 10, indicating that a null hypothesis of a strong instrument is accepted based Stock and Yogo (2002) proposal. These outcomes from the first stage results point to the relevance of the instrument. Consistent with the baseline results, the 2SLS results in both panels show a negative relationship between gambling and savings behaviour. For instance, in column 1 of panel A, the results show that a unit increase in PGSI score reduces the likelihood of saving regularly by 38.5% points. However, the 2SLS results are larger than the baseline results, indicating a downward bias in the baseline results due to endogeneity inherent in the baseline results. Thus, in the presence of endogeneity, the baseline results underestimate the effect of gambling on saving behaviour. The OLS results are biased downwards because the model assumes no endogeneity. However, the IV corrects for endogeneity from all sources, including measurement error, omitted variables bias and reverse causality. For instance, unobserved traits like impulsiveness could influence both gambling and saving, but the OLS model does not account for this. If these unobserved factors bias the OLS estimate downward, IV can give larger, corrected estimates.

**Table 2:** Saving behaviour and gambling-Instrumental variable results

	(1)	(2)	(3)	(4)
	Save regularly	Save regularly	Save leftover	Save leftover
**Panel A**: Traditional 2SLS				
PGSI	-0.382***	-0.375***	-0.215***	-0.273***
	(0.044)	(0.047)	(0.027)	(0.027)
Observations	16,384	15,295	15,500	16,353
Control variables	No	Yes	No	Yes
State fixed effects	Yes	Yes	Yes	Yes
Wave fixed Effects	Yes	Yes	Yes	Yes
***First stage***				
Gambling machines	0.132***	0.099***	0.211***	0.112***
	(0.044)	(0.014)	(0.059)	(0.032)
Cragg-Donald Wald F statistic	31.13	17.93	33.11	26.11
J p-value	0.111	0.189	0.112	0.091
R-squared	0.342	0.452	0.342	0.322
**Panel B**: Lewbel (2012) 2SLS				
PGSI	-0.402***	-0.399***	-0.355***	-0.391***
	(0.051)	(0.051)	(0.049)	(0.043)
Observations	16,384	15,295	15,500	16,353
Control variables	No	Yes	No	Yes
State fixed effects	Yes	Yes	Yes	Yes
Wave fixed Effects	Yes	Yes	Yes	Yes
***First stage***				
Gambling machines	0.212***	0.199***	0.191***	0.291***
	(0.034)	(0.034)	(0.031)	(0.042)
Cragg-Donald Wald F statistic	50.31	34.31	41.02	61.01
J p-value	0.201	0.099	0.092	0.101
R-squared	0.412	0.399	0.412	0.122

Note: We include relevant control variables in all regression. Results in columns 2 and 4 include control variables as in Table 3A in the appendix. Standard errors in parentheses

*** *p* < 0.01, ** *p* < 0.05, * *p* < 0.1

In Table 3, we examine the robustness of our results to different measures of gambling. In panel A, instead of PGSI, we utilize gambling risk as a measure of gambling. As in Sect. 2.2, the gambling risk is on a scale of 1 to 4, where an increasing score suggests a high risk of gambling. Individuals’ participation in gambling increases as the score increases. The results in panel A show a consistent negative relationship between gambling risk and saving behaviour, consistent with baseline results. In panel B of Table 3, we use gambling activities as a measure of gambling. The HILDA survey questionnaire on gambling collects information on about 10 gambling activities. We sum the number of times a respondent participated in any gambling game per day to obtain gambling activities. The results in panel B show a negative relationship between gambling activities and gambling behaviour, indicating that individuals who frequently engage in gambling activities are likely to portray poor saving behaviour. In Panel C of Table 3, we explore gambling expenditure as a measure of gambling because increased gambling expenditure can deter gamblers from saving regularly. To measure gambling expenditure, we use expenses made on gambling activities each month to generate annual gambling expenses and deflate them with income to obtain a share of gambling expenditures. In Panel C, the results show a negative relationship between gambling expenditure and savings behaviour, depicting that gamblers with high gambling expenditure exhibit poor saving behaviour. As gambling expenditure increases, the likelihood of poor saving behaviour also increases, due to both financial constraints and behavioural distortions. Money spent on gambling is money that cannot be saved. The higher the spending, the greater the crowding-out effect on savings.

**Table 3:** Saving behaviour and different measures of gambling

	(1)	(2)	(3)					(4)
	Save regularly	Save regularly	Save leftover					Save leftover
**Panel A: Results based on gambling risk**								
Gambling risk	-0.237***	-0.441***	-0.277***					-0.204***
	(0.046)	(0.172)	(0.093)					(0.097)
Observations	16,384	15,295	15,500					16,353
Control variables	No	Yes	No					Yes
State fixed effects	Yes	Yes	Yes					Yes
Wave fixed Effects	Yes	Yes	Yes					Yes
Year fixed Effects	Yes	Yes	Yes					Yes
R-squared	0.311	0.299	0.192					0.314
**Panel B: Results based on gambling activities**								
Gambling activities	-0.286***	-0.096***	-0.269***					-0.132***
	(0.089)	(0.021)	(0.043)					(0.026)
Observations	16,384	15,295	15,500					16,353
Control variables	No	Yes	No					Yes
State fixed effects	Yes	Yes	Yes					Yes
Wave fixed Effects	Yes	Yes	Yes					Yes
Year Fixed Effects	Yes	Yes	Yes					Yes
R-squared	0.319	0.312	0.292					0.249
**Panel C: Results based on gambling expenditure**								
Gambling expenditure	-0.102***	-0.082***	-0.201***					-0.098***
	(0.034)	(0.011)	(0.046)					(0.020)
Observations	16,384	15,295	15,500					16,353
Control variables	No	Yes	No					Yes
State fixed effects	Yes	Yes	Yes					Yes
Wave fixed Effects	Yes	Yes	Yes					Yes
Year Fixed Effects	Yes	Yes	Yes					Yes
R-squared	0.291	0.212	0.312					0.204

Note: These results are based on instrumental variable strategy. We include relevant control variables in all regression. Results in columns 2 and 4 include control variables as in Table 3A in the appendix. Standard errors in parentheses

*** *p* < 0.01, ** *p* < 0.05, * *p* < 0.1

As discussed in Sect. 2.2, we create four different binary variables to capture gambling risk categories including non-problem gamblers, low-risk gamblers, moderate-risk gamblers and problem gamblers and examine their impact on saving behaviour. The results show that being a low-risk gambler, moderate gambler and problem gambler reduces the likelihood of saving regularly while being a non-problem gambler positively impacts saving regularly. The coefficient of problem gamblers is relatively larger than the others, indicating that gambling adductors portray poor saving behaviour. Non-gamblers are individuals who do not engage in gambling activities and thus are likely to exhibit better-saving behaviour. Overall, the results show that transitioning from a low-risk gambler to a high-risk gambler reduces the likelihood of exhibiting better saving behaviour. This is because high-risk gamblers struggle with self-control and compulsive behaviour. Gambling becomes a habitual or addictive activity, not a planned one. They may gamble even when they cannot afford it, spending savings or income meant for bills, food, or other necessities. Additionally, high-risk gamblers are more likely to chase losses, believing they can win back what they’ve lost. This behaviour leads to ever-increasing bets and higher losses, eating into any disposable income or existing savings (See Table 4).

**Table 4:** Saving behaviour and gambling-effects across different gambling risk spectrums

	(1)	(2)	(3)	(4)
	Save regularly	Save regularly	Save regularly	Save regularly
Non-gambler	0.245***			
	(0.064)			
Low gambler		-0.366***		
		(0.053)		
Mode-gambler			-0.494***	
			(0.067)	
Problem-gambler				-0.524***
				(0.072)
Observations	16,384	15,295	15,500	16,353
R-square	0.284	0.122	0.221	0.222
Control variables	Yes	Yes	Yes	Yes
State fixed effects	Yes	Yes	Yes	Yes
Wave fixed Effects	Yes	Yes	Yes	Yes
Year Fixed Effects	Yes	Yes	Yes	Yes

Note: We include relevant control variables in all regression models. Results in all columns include control variables as in Table 3A in the appendix. Standard errors in parentheses

*** *p* < 0.01, ** *p* < 0.05, * *p* < 0.1

In Table 5, we conduct a sub-sampling analysis across eight different sub-groups to examine the robustness of our results. This is important because the impact of gambling on saving behaviour may differ across these groups. For instance, gender-wise, women’s gambling behaviour is lower than that of men because women have a narrower scope of gambling behaviour (Hraba & Lee, 1996). Hence, we examine the impact of gambling on saving across eight sub-groups. These groups are created based on gender, income grouping, education and age. For gender, we consider men and women, while for households, we consider low- and high-income households. In terms of education, we consider those having below bachelor’s degree and those having a bachelor’s degree or higher. For age, we consider those above 18 years and above and those below 18 years. Across all columns in Table 5, we find a negative relationship between gambling behaviour and saving regularly. However, this effect turns out to be larger for men, low-income households, those having below bachelor’s degree and those aged 18 and above. The results are larger for men because they are more likely to be drawn to competitive, strategic, or high-risk gambling activities. Also, we find the results to be larger for low-income households because low-income households often gamble more frequently than high-income. Low-income individuals often see gambling as a realistic way to escape poverty or improve their financial situation. Finally, one reason why the effect turned out to be larger for people having below a bachelor’s degree is that those with less education often have limited knowledge of financial concepts, such as probability and odds, long-term planning, and the risks of gambling. This lack of knowledge leads to frequent or excessive gambling, which distorts their saving behaviour. Overall, our results show that the intensity of the effect differs across different sub-groups.

**Table 5:** Saving behaviour and gambling-Heterogeneity analysis

	Men	Women	High income	Low income	Having below bachelor’s degree	Having bachelor’s degree or higher	Age below 18 years	Age 18 years and above
	(1)	(2)	(3)	(4)	(5)	(6)	(7)	(8)
	Save regularly	Save regularly	Save regularly	Save regularly	Save regularly	Save regularly	Save regularly	Save regularly
PGSI	-0.234***	-0.119***	-0.095***	-0.111***	-0.193*	-0.107***	-0.087***	-0.198***
	(0.037)	(0.046)	(0.024)	(0.019)	(0.049)	(0.026)	(0.015)	(0.025)
Observations	10,241	10,241	10,241	9,256	9,256	9,256	9,256	10,241
R-square	0.208	0.222	0.212	0.211	0.241	0.211	0.233	0.321
Control variables	Yes	Yes	Yes	Yes	Yes	Yes	Yes	Yes
State fixed effects	Yes	Yes	Yes	Yes	Yes	Yes	Yes	Yes
Wave fixed Effects	Yes	Yes	Yes	Yes	Yes	Yes	Yes	Yes
Year Fixed Effects	Yes	Yes	Yes	Yes	Yes	Yes	Yes	Yes

Note: All regressions include relevant control variables as in Table 3A. Standard errors in parentheses *** *p* < 0.01, ** *p* < 0.05, * *p* < 0.1. PGSI indicates problem gambling severity

### Mediation Analysis

As already discussed in Sect. 1, social capital and locus of control are expected to mediate the relationship between gambling and saving behaviour. Here, we use the two-step approach developed by Baron and Kenny (1986), utilized in recent studies (Koomson et al., 2023; Adabor, 2024a, b; Baako et al., 2024) to test whether social capital and locus of control are possible channels through which gambling impacts saving behaviour. The first step of the Baron and Kenny (1986) approach is that gambling should correlate with the locus of control and social capital. To do this, we estimate the impact of gambling on locus of control and social capital. The results of the impact of gambling on mediators are reported in Table 6. The results show that gambling is positively associated with locus of control but negatively associated with social capital. This suggests that gambling reduces social cohesion and nudges people to be internal on locus of control, in line with existing studies (von der Heiden & Egloff, 2021; Reith & Dobbie, 2011).

**Table 6:** Effect of gambling on locus of control and social capital-first step

	(1)	(3)
	LoC control	Social capital
Gambling	0.155***	-0.058***
	(0.007)	(0.005)
Observations	16,384	15,500
R-square	0.218	0.217
Control variables	No	No
State fixed effects	Yes	Yes
Wave fixed Effects	Yes	Yes

Note: We include relevant control variables in columns 4, 5 and 6 as in Table 3A in the appendix. Standard errors in parentheses. *** *p* < 0.01, ** *p* < 0.05, * *p* < 0.1

The second step is that locus of control and social capital qualify as mediators if after controlling for them in the model (1), reduce the coefficients of gambling or render them insignificant. To test this, we re-estimate the model (1) while controlling for social capital and locus of control in the model. The results for the second step are reported in Table 7. Comparing the coefficient of PGSI in Table 7 to those in Table 1, we observe that the coefficient of PGSI in Table 7 reduces significantly after controlling for social capital, and locus of control. This suggests that social capital, and locus of control are channels for the relationship between gambling and savings behaviour.

**Table 7:** Saving behaviour and gambling-Second step

	(1)	(3)	(4)	(6)
	Save regularly	Save regularly	Save leftover	Save leftover
PGSI	-0.051***	-0.031**	-0.053***	-0.023***
	(0.011)	(0.007)	(0.010)	(0.005)
LoC of control	-0.058***		0.137***	
	(0.012)		(0.039)	
Social capital		-0.108***		-0.089***
		(0.032)		(0.013)
Observations	15,295	15,295	16,353	16,353
R-square	0.088	0.221	0.192	0.189
Control variables	Yes	Yes	Yes	Yes
State fixed effects	Yes	Yes	Yes	Yes
Wave fixed Effects	Yes	Yes	Yes	Yes
Year Fixed Effects	Yes	Yes	Yes	Yes

Note: All regressions include relevant control variables as in Table 3A. Standard errors in parentheses *** *p* < 0.01, ** *p* < 0.05, * *p* < 0.1. PGSI indicates problem gambling severity

## Conclusion and Policy Implications

Gambling is a significant part of Australian culture and economy, with one of the highest per capita gambling rates in the world. Australians spend billions annually on various forms of gambling, including pokies (slot machines), sports betting, lotteries, and casinos (Baako et al., 2024; Letts, 2018). While it contributes substantially to government revenue, gambling has also raised concerns due to its social and financial impact, particularly the high rates of problem gambling and related harm. As a result, gambling is now considered a major public issue in Australia, yielding a significant policy interest. This has led to a growing body of literature examining the implications of gambling in Australia to guide the implementation of gambling policies to reduce gambling participation and the harm associated with it. This study adds to this growing body of research and policy by examining the relationship between gambling and saving behaviour. Also, the study unearths the potential channels through which gambling impacts saving behaviour.

The main finding is that an increase in gambling participation is associated with poor savings behaviour. Thus, gamblers are more likely to exhibit poor saving behaviour. However, the size of this effect was found to differ across eight different sub-groups. Nonetheless, we found a consistent negative relationship between gambling and savings behaviour after employing alternative measures of gambling including gambling risk, gambling activities and gambling expenditure. The results based on gambling risk status show that shifting from low-risk category to high-risk category is linked with an even higher reduction in the likelihood of saving regularly. Finally, the mediation results show that social capital and locus of control are the mechanisms through which gambling transmits to poor saving behaviour.

Many adult Australians are spending more on gambling, leaving no room for them to manage their income by setting aside a portion for future use. Thus, many Australians are not practicing good saving behaviour because of the increased rate of gambling participation. Good saving behaviour is imperative because it helps individuals to smooth their consumption over time, accumulate assets, invest and improve overall well-being (Lydall, 1955; Brown et al., 2021). To improve saving behaviour, the Australian governments need to implement strategies to reduce the rate at which Australians participate in gambling. Thus, interventions and policies aimed at decreasing participation and access to sports betting, bingo, lottery, and casino games will enable Australians to have control and manage their income effectively by setting some aside for future use, enhancing their saving behaviour. Precisely, the government needs to implement educational programs that specifically address the impact of gambling on long-term financial well-being and the importance of saving. These could be integrated into schools, vocational training, and community centers. This will improve financial literacy to counteract impulsive financial decisions and help individuals recognize the opportunity costs of gambling. Additionally, the government should enforce systems where gamblers must set gambling spending and time limits before playing. Finally, it is important to limit gambling advertising during sporting events, late-night hours, and on digital platforms frequented. This will reduce exposure to gambling cues, which can trigger impulsive betting and lower savings. Since problem gamblers are gambling adductors, much effort or strategies should be geared toward helping problem gamblers to progressively transition to lower risk status over time.

The mediating role of social capital indicates that efforts to improve social cohesion can weaken the adverse impact of gambling on saving behaviour. Precisely, it is important to strengthen social ties and shared financial responsibility via establishing community savings groups (e.g., rotating savings and credit associations or peer-led “money circles”) in areas with high gambling activity. These groups can foster mutual accountability, reinforce saving behaviour, and reduce financial isolation, which can weaken the grip of gambling. The government should partner with local cultural, religious, and community organizations to deliver tailored workshops on saving and the risks of gambling. When financial education is embedded in familiar social settings, it’s more likely to be accepted, internalized, and shared within networks.

Next, the mediating role of locus of control indicates that teaching people to develop positive self-control beliefs or believing in one’s ability to deal with issues can reduce gambling participation, extending to improving saving behaviour. Precisely, designing financial literacy initiatives that go beyond knowledge delivery to include goal setting, budgeting with feedback loops, and personal agency-building exercises can teach people how to act on financial knowledge, help them feel in control of their outcomes, and reduce reliance on gambling as a quick fix. Additionally, the government should implement early interventions to help young people develop a sense of agency before gambling behaviours begin. These interventions include designing modules in school curricula that teach children how their choices affect future outcomes through simple games, simulations, and financial planning exercises. This will help young people develop an internal locus of control at an early age.

Our findings also have some macroeconomic implications. First, gambling losses directly erode disposable income that could otherwise be directed toward wealth-building activities, including savings deposits, investing in property, and acquiring financial assets. This diversion of resources slows capital accumulation, especially for low- and middle-income households, where gambling often consumes a larger proportion of income. Over time, this contributes to greater wealth inequality, as these households fall further behind in asset ownership and intergenerational wealth transfer. On a broader scale, persistent gambling losses can negatively affect a country’s aggregate savings rate. As more income is channelled into gambling, less is saved in bank accounts or long-term instruments. Gambling-related financial strain may lead to credit reliance, lowering net savings, and increasing vulnerability. This reduces funds available for domestic investment and increases greater reliance on foreign capital for investment and infrastructure.

## Limitations and Areas for Further Research

This study employed a quantitative approach, which may not fully capture the complex motivations and lived experiences behind gambling and saving behaviours. Future studies could incorporate qualitative methods, such as interviews or focus groups, to gain deeper insights into how individuals perceive the impact of social connections and personal control on their financial decision-making and gambling behaviour.

Other psychological factors, such as financial literacy, impulsivity, stress, or peer influence, may also mediate or moderate the relationship between gambling and saving behaviour. Including a broader set of variables in future models could provide a more comprehensive understanding of the behavioural mechanisms at play.

Furthermore, although the mediation analysis offers valuable policy insights, its impact could be strengthened by quantifying the direct and indirect effects of gambling on savings behaviour. Achieving this would require advanced structural equation modelling, an approach beyond the scope of this study, but a promising avenue for future research.

In summary, while this study contributes to the understanding of the interplay between gambling, saving behaviour, and psychosocial mediators, future research addressing these limitations will help build a more robust and contextually nuanced body of knowledge.

## Appendix

**Table 1A:** Nine questions were used to derive the problem gambling severity index

Thinking about the last twelve months	Never (0)	Sometimes (1)	Most of the time (2)	Almost Always (3)
1. Have you felt guilty about the way you gamble or what happens when you gamble?				
2. Have you bet more than you could really afford to lose?				
3. Has your gambling caused any financial problems for you or your household?				
4. Have you needed to gamble with larger amounts of money to get the same feeling of excitement?				
5. Have people criticized your betting or told you that you had a gambling problem, regardless of whether you thought it was true?				
6. When you gambled, did you go back another day to try and win back the money you lost?				
7. Has gambling caused you any health problems, including stress or anxiety?				
8. Have you borrowed money or sold anything to get money to gamble?				
9. Have you felt that you might have a problem with gambling?				

**Table 2A:** Description and summary statistics for all the variables

Variable		Mean	Std. Dev.
***Outcome variables***			
Save regularly	A binary variable set equal to one if respondents save regularly	0.497	0.448
Save leftover	A binary variable set equal to one if respondents save leftover		
***Key independent variables***			
PGSI score	PGSI gambling scale based on 9 items in Table 1 with a maximum score of 27	0.326	1.622
Gambling expenditure	Total expenditure on gambling as a share of income in dollars per month	5.619	1.376
Gambling activity	The number of gambling activities respondents participated in the past 12 months	1.159	3.337
Gambling risk	1–4 gambling scale based on PGSI score, 1 means “problem gambler”, 2 means “low-risk gambler”, 3 means “moderate risk gambler” and 4 means “problem gambler”.	1.135	0.492
***Control variables***			
Age	Age of respondent in years	51.731	14.40
Male	The dummy variable is set equal to 1 if the respondent is a male	0.584	0.492
Household income	The continuous variable for the log is the average income of the household	7.681	3.278
De facto	The dummy variable is set equal to if the respondent is de facto	0.139	0.346
Separated	The dummy variable is set equal to if the respondent is separated	0.043	0.202
Widowed	The dummy variable is set equal to 1 if the respondent is widowed	0.078	0.268
Divorced	The dummy variable is set equal to1 if the respondent is divorced	0.100	0.300
Household size	Number of individuals living in a household	2.251	0.980
Never married	The dummy variable is set equal to1 if the respondent is never married	0.203	0.402
Long-term health condition	The dummy variable set equal to if the respondent has a long-term health condition	0.256	0.436
Certificate	The dummy variable set equal to 1 if the respondent’s highest level of education achieved is the certificate I, II, II, and IV.	0.244	0.429
Diploma	The dummy variable set equals 1 if the respondent’s highest level of education achieved is a diploma or advanced diploma.	0.097	0.297
Bachelor	The dummy variable set equals to 1 if the respondent’s highest level of education achieved is Bachelor’s or honours.	0.148	0.355
Postgraduate	The dummy variable set equals 1 if the respondent’s highest level of education achieved is a master’s or doctorate.	0.058	0.234
Total children ever had	Total number of children born to the respondent	1.788	1.578
Homeowner	The dummy variable is set equal to 1 if the respondent owns a home	0.630	0.482
Unemployed	The dummy variable is set equal to 1 if the respondent is unemployed	0.026	0.159

*** *p* < 0.01, ** *p* < 0.05, * *p* < 0.1

**Table 3A:** Saving behaviour and gambling-Full baseline results

	(1)	(2)	(3)	(4)
	Save regularly	Save regularly	Save leftover	Save leftover
PGSI	-0.125***	-0.106***	-0.072***	-0.069***
	(0.014)	(0.014)	(0.010)	(0.010)
Male		-0.043		-0.139***
		(0.044)		(0.039)
Age		0.002		0.002
		(0.002)		(0.001)
Household size		-0.219*		-0.147
		(0.113)		(0.107)
Bachelor or honours		0.282***		0.167**
		(0.098)		(0.093)
Diploma		0.412***		0.291***
		(0.103)		(0.097)
Certificate		0.205***		0.175***
		(0.052)		(0.037)
Postgraduate		0.163***		0.115**
		(0.045)		(0.035)
Household income (in log)		0.421***		0.228**
		(0.096)		(0.089)
De facto		-0.108		-0.036
		(0.067)		(0.061)
Separated		-0.351***		-0.104
		(0.105)		(0.085)
Divorced		-0.136*		-0.152**
		(0.073)		(0.064)
Widowed		-0.425***		-0.271***
		(0.097)		(0.085)
Never married		0.222***		0.098*
		(0.067)		(0.059)
Long-term health condition,		-0.427***		-0.238***
		(0.051)		(0.044)
Number of children ever had		-0.149***		-0.048***
		(0.016)		(0.014)
Homeowner		0.319***		0.274***
		(0.048)		(0.043)
Unemployed		-0.792***		-0.631***
		(0.130)		(0.107)
Observations	16,384	15,295	15,500	16,353
Year Fixed Effects	Yes	Yes	Yes	Yes
State Fixed Effects	Yes	Yes	Yes	Yes
Wave fixed Effects	Yes	Yes	Yes	Yes

## Data Availability

The author does not have permission to share the Household, Income, and Labour Dynamics in Australia (HILDA) Survey data.
